# Reduced cerebrospinal fluid motion in patients with Parkinson’s disease revealed by magnetic resonance imaging with low b-value diffusion weighted imaging

**DOI:** 10.1186/s12987-024-00542-8

**Published:** 2024-05-09

**Authors:** Gabriela Pierobon Mays, Kilian Hett, Jarrod Eisma, Colin D. McKnight, Jason Elenberger, Alexander K. Song, Ciaran Considine, Wesley T. Richerson, Caleb Han, Adam Stark, Daniel O. Claassen, Manus J. Donahue

**Affiliations:** 1 Department of Neurology, Vanderbilt University Medical Center, Nashville, TN USA; 2 Department of Radiology and Radiological Sciences, Vanderbilt University Medical Center, Nashville, TN USA; 3 Department of Psychiatry and Behavioral Sciences, Vanderbilt University Medical Center, Nashville, TN USA

**Keywords:** Glymphatic, Suprasellar cistern, DWI, Cerebrospinal fluid, Parkinson’s, α-synuclein, Choroid plexus

## Abstract

**Background:**

Parkinson’s disease is characterized by dopamine-responsive symptoms as well as aggregation of α-synuclein protofibrils. New diagnostic methods assess α-synuclein aggregation characteristics from cerebrospinal fluid (CSF) and recent pathophysiologic mechanisms suggest that CSF circulation disruptions may precipitate α-synuclein retention. Here, diffusion-weighted MRI with low-to-intermediate diffusion-weightings was applied to test the hypothesis that CSF motion is reduced in Parkinson’s disease relative to healthy participants.

**Methods:**

Multi-shell diffusion weighted MRI (spatial resolution = 1.8 × 1.8 × 4.0 mm) with low-to-intermediate diffusion weightings (*b*-values = 0, 50, 100, 200, 300, 700, and 1000 s/mm^2^) was applied over the approximate kinetic range of suprasellar cistern fluid motion at 3 Tesla in Parkinson’s disease (*n* = 27; age = 66 ± 6.7 years) and non-Parkinson’s control (*n* = 32; age = 68 ± 8.9 years) participants. Wilcoxon rank-sum tests were applied to test the primary hypothesis that the noise floor-corrected decay rate of CSF signal as a function of *b*-value, which reflects increasing fluid motion, is reduced within the suprasellar cistern of persons with versus without Parkinson’s disease and inversely relates to choroid plexus activity assessed from perfusion-weighted MRI (significance-criteria: *p* < 0.05).

**Results:**

Consistent with the primary hypothesis, CSF decay rates were higher in healthy (*D* = 0.00673 ± 0.00213 mm^2^/s) relative to Parkinson’s disease (*D* = 0.00517 ± 0.00110 mm^2^/s) participants. This finding was preserved after controlling for age and sex and was observed in the posterior region of the suprasellar cistern (*p* < 0.001). An inverse correlation between choroid plexus perfusion and decay rate in the voxels within the suprasellar cistern (Spearman’s-*r*=-0.312; *p* = 0.019) was observed.

**Conclusions:**

Multi-shell diffusion MRI was applied to identify reduced CSF motion at the level of the suprasellar cistern in adults with versus without Parkinson’s disease; the strengths and limitations of this methodology are discussed in the context of the growing literature on CSF flow.

## Introduction

Parkinson’s disease (PD) is a progressive neurodegenerative disorder with symptoms of cognitive and motor decline, and pathologic aggregation and accumulation of α-synuclein protofibrils [1]. As α-synuclein is distributed throughout the cerebrospinal fluid (CSF), and the topography of α-synuclein clearance and aggregation may contribute to disease propagation in PD, neurofluid motion assessments in PD may be useful to develop new pathophysiologic models of disease progression [2, 3].

CSF, produced within the choroid plexus complexes, is the primary medium for clearance of waste products and proteins and traverses traditional bulk and likely recently identified perivascular pathways [4, 5]. Arterial pulsatility is believed to be fundamental to anterograde fluid movement [6], whereas fluid transport along the parasagittal dural spaces and cranial nerves has been proposed to have egress relevance [7, 8]. Major intracranial arteries are bounded by free CSF at the level of the suprasellar cistern, and as such, quantitative imaging of CSF motion within the suprasellar cistern may provide one relevant marker of CSF circulation, and this motion could have an association with CSF production or flow across different brain regions.

In vivo assessments of this motion have been difficult given limited imaging approaches for quantifying CSF and other fluid motion non-invasively in vivo. Bulk CSF flow at the level of the cerebral aqueduct can be interrogated using quantitative phase contrast magnetic resonance imaging [8–10], and perivascular motion can be estimated qualitatively from *T*_2_-weighted MRI or *T*_1_-weighted MRI before and after intrathecal contrast agent administration [11]. However, such imaging of perivascular motion generally does not provide quantitative values and additionally suffers from the caveats that intrathecally administered contrast is contraindicated for research studies in most centers and may not fully reflect in vivo kinetics.

To continue to address these limitations, we implemented a multi-shell diffusion weighted imaging (DWI) magnetic resonance imaging (MRI) protocol and analysis approach, which incorporates a range of diffusion weightings over the approximate kinetic range of CSF motion within the suprasellar cistern. This approach, which has previously been proposed in healthy adults using categorical scoring of signal attenuation for increasing diffusion weighting [12], is applied here to (i) investigate differences in quantification procedures, (ii) demonstrate the corresponding sensitivity to regions with known differences in CSF motion and (iii) quantify relationships between CSF motion and choroid plexus perfusion in older adults with and without PD. The suprasellar cistern was chosen as a site of interest as it is a fluid filled cavity that contains major arteries and cranial nerves, and motion within this cistern can provide a potential proxy of CSF motion through the cerebrum; furthermore, as the suprasellar cistern is largely devoid of cerebral capillaries, contrast confounds on diffusion imaging from slow arteriolar or capillary flow are reduced. The hypothesis to be investigated is that CSF motion within the suprasellar cistern of patients with PD is reduced relative to age-matched controls. A secondary hypothesis is that markers of CSF production activity are related to the reduced CSF movement within the suprasellar cistern. Findings are discussed in the context of the growing literature on CSF circulation and neurodegeneration.

## Methods

### Demographics

Adult participants with and without Movement Disorder Society clinical criteria for PD [13] with no other history of neurological disease were recruited and provided informed consent for this prospective Institutional Review Board (IRB)-approved study. Disease duration as well as the Unified Parkinson’s disease rating scale (UPDRS) [14] were both recorded in all participants. Exclusion criteria were: independent neurological or psychiatric condition including but not limited to Alzheimer’s disease, multiple sclerosis, prior overt stroke, schizophrenia, or bipolar disorder; flow-limiting cervical or intracranial stenosis (i.e., stenosis > 70%) of the internal carotid arteries, vertebral or basilar arteries, or first segment of the anterior, middle, or posterior cerebral arteries; independent condition expected to lead to death within one year; and any contraindication to 3 Tesla MRI. Presence of non-specific diffuse white matter lesions was not an exclusion criterion, as these lesions become more prevalent with aging, and we sought our control cohort to be generalizable and representative. Parkinsonism medications (e.g., dopamine agonist and dopamine replacement therapies) were withheld for 16-hours prior to scanning. Exclusion criteria were identical for control participants and additionally expanded to include positive mental health negative for neuro-psychiatric conditions and no complaints of prior cognitive issues.

### Non-imaging assessments

PD and control participants underwent cognitive screening with the Montreal Cognitive Assessment (MoCA) [15] and brief neuropsychological test examination with the Repeatable Battery for the Assessment of Neuropsychological Status (RBANS) [16]. PD participants were additionally screened by a board-certified neurologist (DOC) to ensure PD criteria were met [14].

### Experiment

Participants were scanned at 3 Tesla (Philips Healthcare, Best, The Netherlands) using body coil radiofrequency transmission and SENSE phased-array 32-channel reception. As it has been shown that CSF production activity is coupled to circadian rhythms [17], we imaged consistently between 7:00 AM and 11:00 AM.

To characterize tissue and vascular health, a standard non-contrast head protocol was applied: (i) 3D *T*_1_-weighted magnetization-prepared-rapid-gradient-echo (TR = 8.1 ms; TE = 3.7 ms; field-of-view = 256 × 180 × 150 mm; slices = 150; spatial resolution = 1.0 × 1.0 × 1.0 mm; duration = 4:32), (ii) 2D *T*_2_-weighted FLuid-Attenuated-Inversion-Recovery (FLAIR) turbo-spin-echo (TR = 11s; TE = 120 ms; TI = 2,800 ms; field-of-view = 230 × 184 × 144 mm; slices = 29; spatial resolution = 0.57 × 0.57 × 4.0 mm; duration = 1:39), (iii) 3D *T*_2_-weighted turbo-spin-echo (TR = 2,500 ms; TE = 331 ms; field-of-view = 250 × 250 × 189 mm; slices = 242; spatial resolution = 0.78 × 0.78 × 0.78 mm; duration = 4:08), (iv) 2D diffusion-weighted spin-echo with single-shot echo-planar imaging readout (TR = 2,926 ms; TE = 83 ms; field-of-view = 229 × 229 × 139 mm; slices = 28; spatial resolution = 1.8 × 1.8 × 4.0 mm; *b*-value = 1000 s/mm^2^; duration = 0:58), and (v) 3D time-of-flight magnetic resonance angiography (TR = 23 ms; TE = 3.5 ms; field-of-view = 200 × 200 × 84 mm; slices = 120; spatial resolution = 0.39 × 0.39 × 0.70 mm; duration = 4:07). These images and angiograms were used to ensure inclusion criteria were met.

For functional assessment of CSF motion and choroid plexus perfusion, multi-shell DWI and pseudo-continuous arterial spin labeling (pCASL), respectively, were applied in sequence. 2D echo-planar-imaging DWI with common field-of-view = 230 × 230 × 139 mm, diffusion directions = 6, slices = 28, spatial resolution = 1.8 × 1.8 × 4.0 mm, TE = 83 ms, and TR = 2,539 ms were acquired for seven *b*-values which were randomized in order (*b*-values = 0, 50, 100, 200, 300, 700, and 1000 s/mm^2^), similar to a recent qualitative report of signal decay using the same principles [12]. The randomization was performed simply to reduce any potential system effects from drift or gradient heating over the course of the protocol. To evaluate choroid plexus perfusion, a recently-reported pCASL approach tested for reproducibility [9, 18] to choroid plexus perfusion was used with TR = 4,550 ms, TE = 11 ms, post-label delay = 2,000 ms and with the labeling plane (0.5 ms Hanning-windowed pulses; pulse-train duration = 1800 ms) placed proximal to anterior and posterior choroidal arteries (which supply the choroid plexus), approximately 100 mm proximal to the corpus callosum. A TR = 15s acquisition with identical geometry and spin labeling removed was acquired for equilibrium magnetization (M_0_) determination.

### Analysis

Anatomical imaging and angiography scans were reviewed by a board-certified radiologist (CDM; experience = 9 years) to ensure that no exclusion criteria were met. Clinical history was reviewed by a board-certified neurologist (DOC; experience = 16 years) to ensure participants met healthy control or PD criteria. A board-certified neuropsychologist (CMC; experience = 5 years) reviewed all cognitive screening and neuropsychological test data to ensure stated inclusion criteria regarding cognition were met.

Choroid plexus segmentations were generated from a previously-published [9, 19] fully convolutional neural network with 3-D U-Net architecture utilizing co-registered *T*_1_-weighted and *T*_2_-weighted FLAIR images in 1 mm isotropic MNI152 space. All registrations were performed using the Advanced Normalization Tools (ANTs) nonlinear registration tools [20]. Choroid plexus perfusion was quantified within the atria of the lateral ventricles following previously reported procedures [9, 19] but expanded to include recent literature reports of choroid plexus *T*_1_ and bolus arrival time [9]. Briefly, the source pCASL control and label images were surround subtracted [21], slice delay corrected, averaged, and fit to the third component of the Buxton three-stage kinetic model [22]:

1 $$\varDelta M\left(t\right)=2\alpha {M}_{0,blood}f{T}_{1,app}{\cdot e}^{\frac{-{\Delta }t}{{T}_{1,blood}}}\cdot {e}^{\frac{-\left(t-\tau -{\Delta }t\right)}{{T}_{1,app}}}\cdot (1-{e}^{\frac{-\tau }{{T}_{1,app}}})$$

with,

2 $$\frac{1}{{T}_{1,app}}=\frac{1}{{T}_{1,choroid\ plexus}}+\frac{f}{\lambda }$$

for blood-brain partition coefficient (λ) = 0.9 mL blood/g brain, labeling efficiency from the dual-background suppressed sequence (α) = 0.8, measured equilibrium blood water magnetization = M_0,blood_, labeling duration τ = 1,800 ms, post-label delay *t* = 2,000 ms, choroid plexus bolus arrival time Δt = 1,240 ms [23], *T*_1,choroid plexus_=2,500 ms [24], and *T*_1,blood_ = 1,624 ms [25]. Note that the simplified International Society for Magnetic Resonance in Medicine Perfusion Study Group [26] kinetic model was not used as this assumes that tissue (i.e., choroid plexus) *T*_1_ is similar to blood *T*_1_, and this is a poor approximation in choroid plexus. The processed perfusion maps were co-registered to the *T*_1_-weighted images using the ANTs non-linear registration tools [20], and the choroid plexus segmentation from the machine learning model was applied to the perfusion maps to calculate the mean perfusion values in the choroid plexus of the lateral ventricles. A minimum threshold of 10 mL/100 g/min was used for perfusion quantification to ensure that all values were above the noise floor.

All isotropic diffusion scans were aligned to the ICBM-MNI 152 symmetric proton density template [27] using the diffusion map acquired using the highest signal-to-noise ratio *b* = 50 s/mm^2^ scan. Transformations were subsequently applied to all other *b*-values. Spatial diffeomorphism transformations were estimated using the symmetric normalization method [28] provided in ANTs [20]. Maps of CSF motion within the suprasellar cistern were calculated from the multi-shell DWI scan. Trace images were corrected for bulk head motion [20] and signal was fit as a function of *b*-value, including a term for the noise floor (*N*_floor_) [29]:

3 $$S(b) = {S_0}\,\exp ( - b\, \cdot D) + {N_{{\rm{floor}}}}$$

where *S* is the signal as a function of *b*-value, *D* is the apparent diffusion coefficient for water, and *S*_0_ is signal in the absence of diffusion-sensitizing gradients, and *b* is the *b*-value=(γGδ)^2^Δ for the narrow pulse approximation with gradient amplitude *G*, the diffusion time *Δ*, and gradient pulse width, *δ* [30]. Note that in this fitting procedure, which is analogous to the calculation of the apparent diffusion coefficient (ADC), larger decay rates (*D*) reflect faster decay across the *b*-value range and as such higher CSF motion. To demonstrate the impact of including the N_floor_, we also repeated fitting without this term for completeness.

Finally, total gray matter, total white matter, and ventricular CSF masks were calculated from *T*_1_-weighted scans using AssemblyNet [31], a deep-learning software specializing in the segmentation of brain structures.

### Statistical considerations and hypothesis testing

Descriptive statistics, including means and standard deviations of continuous imaging parameters, as well as percentages and frequencies of categorical parameters, were calculated. Given the sample size (e.g., 27–32 participants per group), it is difficult to estimate the data distribution exactly and as such non-parametric tests were used. A Wilcoxon rank sum test was applied to evaluate differences in continuous measures, whereas a chi-squared test was applied to evaluate differences in categorical measures. First, root-mean-squared error (RMSE) values for fitting were calculated in an ablation fitting routine (e.g., fitting to all *b*-values or a subset of *b*-values) to understand whether high *b*-value data biased fit results given low fluid signal for this high level of diffusion suppression. Here, the Rician-corrected high *b* = 1000 s/mm^2^ magnitude image was used as an estimate of the noise in the suprasellar cistern. For the primary analysis of interest, we utilized the noise floor fitting to all *b*-values (e.g., Eq. 3) and corresponding fit statistics including R^2^, Akaike information criterion (AIC), and residuals were calculated. Next, to ensure that the multi-shell DWI protocol was sensitive to known regions with different motion, voxel-wise decay rate (*D*) values in ventricular CSF, total gray matter, and total white matter were contrasted across all participants. Summary statistics which describe normative values in each region for PD and control participants were recorded. To test the overarching hypothesis that suprasellar cistern CSF motion was reduced in PD relative to control participants, voxel-wise analysis within the suprasellar cistern was conducted using the computational anatomy toolbox (CAT12). To limit overfitting issues, uncorrected cluster of voxels were defined with a conservative statistical threshold set to corrected *p* = 0.10 at a voxel level. Significant voxels within the cavity of the suprasellar cistern vs. optic nerve were considered separately: values within the detected clusters were averaged in the two different sub-regions. For these values, a Wilcoxon rank sum test was applied between mean rates for each cohort; separate linear regression analyses which modeled the dependence of the decay rate on (i) cohort, (ii) age, and (iii) sex were performed. Finally, to understand whether reduced motion was related to elevated choroid plexus activity, Spearman rank-order correlation testing was applied using the decay rate as the dependent variable and choroid plexus perfusion as the independent variable, separately for identified clusters in the suprasellar cistern and optic nerve. As an exploratory analysis, separate regression analyses were performed to evaluate any dependence of (i) UPDRS (motor function) or (ii) MoCA (cognitive function) on the independent variable of decay rates within the suprasellar cistern region in PD participants. For all comparisons, significance was defined as two-sided *p* < 0.05.

### Data sharing

Source imaging and demographic data will be made available to investigators with appropriate Collaborative of Institutional Training Initiative *Responsible Conduct of Human Research* and *Good Clinical Practice* training upon request.

## Results

Participants are summarized in Table 1 depicting 59 enrolled participants, including 27 with PD (age = 66.0 ± 6.8 years) and 32 non-PD controls (age = 67.6 ± 9.0 years). Participants between groups were matched for age (*p* = 0.465) and no participants had evidence of vasculopathy or met radiological exclusion criteria. One control with atypical cognitive screen (MoCA < 24) [32] was determined to demonstrate clinically significant performance deficits on neuropsychological test examination (1 + score < 2 standard deviations, or, 2 + scores < 1.5 standard deviations) [33] and was considered separately in a sub-analysis. Five controls with atypical cognitive screens (MoCA = 22–23) were determined to demonstrate performance on neuropsychological test examination within normal limits, and thus were included in analyses.

**Table 1:** Participant demographics and measures of suprasellar cistern motion and choroid plexus perfusion

	Control	Parkinson’s disease	*p*-value
N	32	27	
Age (years)	67.6 ± 9.0	66.0 ± 6.8	0.465
Unified Parkinson’s Disease Rating Scale (UPDRS)	n / a	32.4 ± 12.5	-
Montreal Cognitive Assessment (MoCA)	26.5 ± 2.8	24.3 ± 3.9	0.026*
Vasculopathy present (percent)	0 ± 0	0 ± 0	1.000
*Noise-modeled fit*			
Ventricle decay rate, *D* (mm^2^/s)	0.00253 ± 0.00019	0.00267 ± 0.00028	0.027*
Total gray matter decay rate, *D* (mm^2^/s)	0.00209 ± 0.00009	0.00211 ± 0.00016	0.442
Total white matter decay rate, *D* (mm^2^/s)	0.00210 ± 0.00010	0.00212 ± 0.00015	0.498
Suprasellar cistern decay rate, *D* (mm^2^/s)	0.00673 ± 0.00213	0.00517 ± 0.00110	0.001**
Optic nerve decay rate, D (mm^2^/s)	0.00453 ± 0.00249	0.00363 ± 0.00108	0.069
Choroid plexus perfusion (ml/100 g/min)	32.7 ± 6.1	32.8 ± 8.2	0.932

n / a: not applicable. * significant at two-sided *p* < 0.05 prior to multiple comparisons corrections. ** (bold) significant at stated criteria after multiple comparisons corrections

**Fig. 1:**
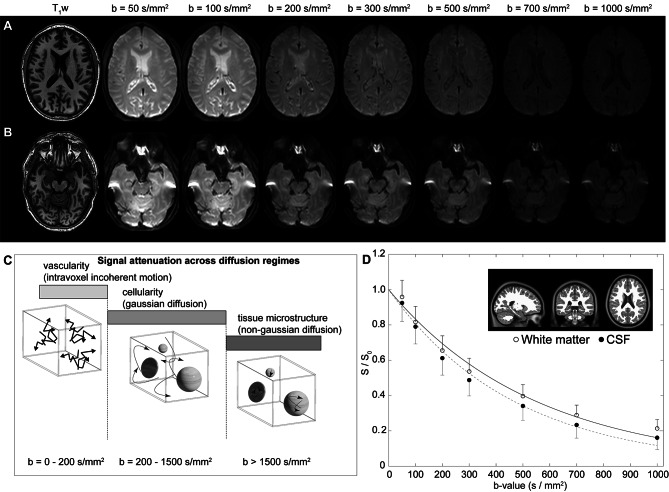
Multi-shell diffusion weighted imaging (DWI) using low-to-intermediate *b*-values. (**A**) Two slices at the level of the lateral ventricles (**A**) and suprasellar cistern (**B**) for a representative participant (age = 77 years; sex = male) are shown. (**C**) The approximate regime of physiological sensitivity for increasing *b*-values, whereby low *b*-values < 200 s/mm^2^ have known sensitivity to vascular structures and intravoxel incoherent motion, intermediate *b*-values of approxiamtely 200–1500 s/mm^2^ are sensitive to cellularity in the regime of gaussian diffusion, and high *b*-values above 1500 s/mm^2^ are most sensitive to non-gaussian diffusion and tissue microstructure assessments. (**D**) Example decay curves as a function of low-to-intermediate *b*-value in the transition range of intravoxel incoherent motion and gaussian diffusion demonstrate differences in cerebrospinal fluid and tissue. Error bars across the region shown in the insert are depicted as one-sided for clarity

Figure 1A-B shows representative DWI in a participant as a function of increasing *b*-value, whereby faster flowing fluid is suppressed in all *b*-values and slower flowing fluid is suppressed in larger *b*-values only. Figure 1C summarizes the ranges of diffusion for different types of motion, including intravoxel incoherent motion, gaussian diffusion, and non-gaussian diffusion, and the relevant approximate diffusion weightings for each regime. Figure 1D shows accelerated signal decay at the level of CSF relative to tissue. It should be noted that since parenchymal motion in this range may be attributable to capillary flow, interstitial flow, or perivascular fluid flow, we focused our analysis on CSF motion within the suprasellar cistern.

**Fig. 2:**
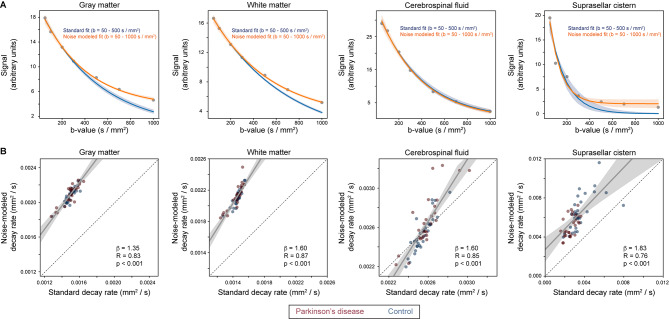
Fitting procedures. (**A**) Signal decay, along with exponential fitting, across a range of regions and tissue structures. Given higher motion for neurofluids relative to gray and white matter, fitting and confidence intervals are shown in blue for the lower *b*-values (e.g., 5–500 s/mm^2^) without the noise floor modeled as well as separately in orange for all *b*-values by including the noise floor term (e.g., Eq. 3). Across all tissue types it can be appreciated that by modeling the noise floor, the goodness of fit improves, as expected, for the range of *b*-values. Quantitative fitting statistics are summarized in Table 2. (**B**) The quantitative relationship for decay rates when the noise floor is fit versus when it is excluded. Solid lines depict best fit lines whereas dashed lines depict lines of unity. In all subsequent analyses, the fitting procedure that incorporated the noise floor modeling was used

Figure 2A demonstrates signal decay, along with exponential fitting, across a range of regions and tissue structures, including (i) gray matter, (ii) white matter, (iii) total cerebrospinal fluid, and (iv) suprasellar cistern cerebrospinal fluid. Given higher motion for neurofluids relative to gray and white matter, fitting and confidence intervals are shown for the lower *b*-values (e.g., 5–500 s/mm^2^) without the noise floor modeled as well as separately for all *b*-values by including the noise floor term (e.g., Eq. 3). Across all tissue types it can be appreciated that by modeling the noise floor, the goodness of fit improves, as expected, for the range of b-values. In all subsequent analyses and figures, results are reported when the noise floor is modeled (e.g., Eq. 3). Figure 2B demonstrates the quantitative relationship for decay rates when the noise floor is fit versus when it is excluded.

**Table 2:** Comparison of diffusion coefficients in different tissue types when the standard model is fit to the signal decay versus when the noise floor is additionally modeled. We evaluated, in the above regions-of-interest, the two models using the residual, coefficient of determination criteria (R^2^) and the Akaike information criterion (AIC). We observed that in all regions considered, both models have satisfactory fitting, with high R^2^ (ranging from 0.95 to 1.0) and low AIC (ranging from 69 to 116). However, it is noteworthy that the noise-modeled decay obtains overall better fitting values when R^2^ and residuals are considered; larger AIC likely results from the higher number of parameters as AIC penalizes the increase of model complexity. Quantitative fit values are summarized in Table 1

	Standard modeled decay	Noise modeled decay
	Suprasellar cistern	
Coefficient of determination (R^2^)	0.93	0.97
Akaike information criterion (AIC)	79	116
Residual	597	402
	Cerebrospinal fluid	
Coefficient of determination (R^2^)	1.00	1.00
Akaike information criterion (AIC)	69	105
Residual	207	196
	Gray matter	
Coefficient of determination (R^2^)	0.95	1.00
Akaike information criterion (AIC)	75	89
Residual	367	71
	White matter	
Coefficient of determination (R^2^)	0.97	1.00
Akaike information criterion (AIC)	71	76
Residual	246	31

**Fig. 3:**
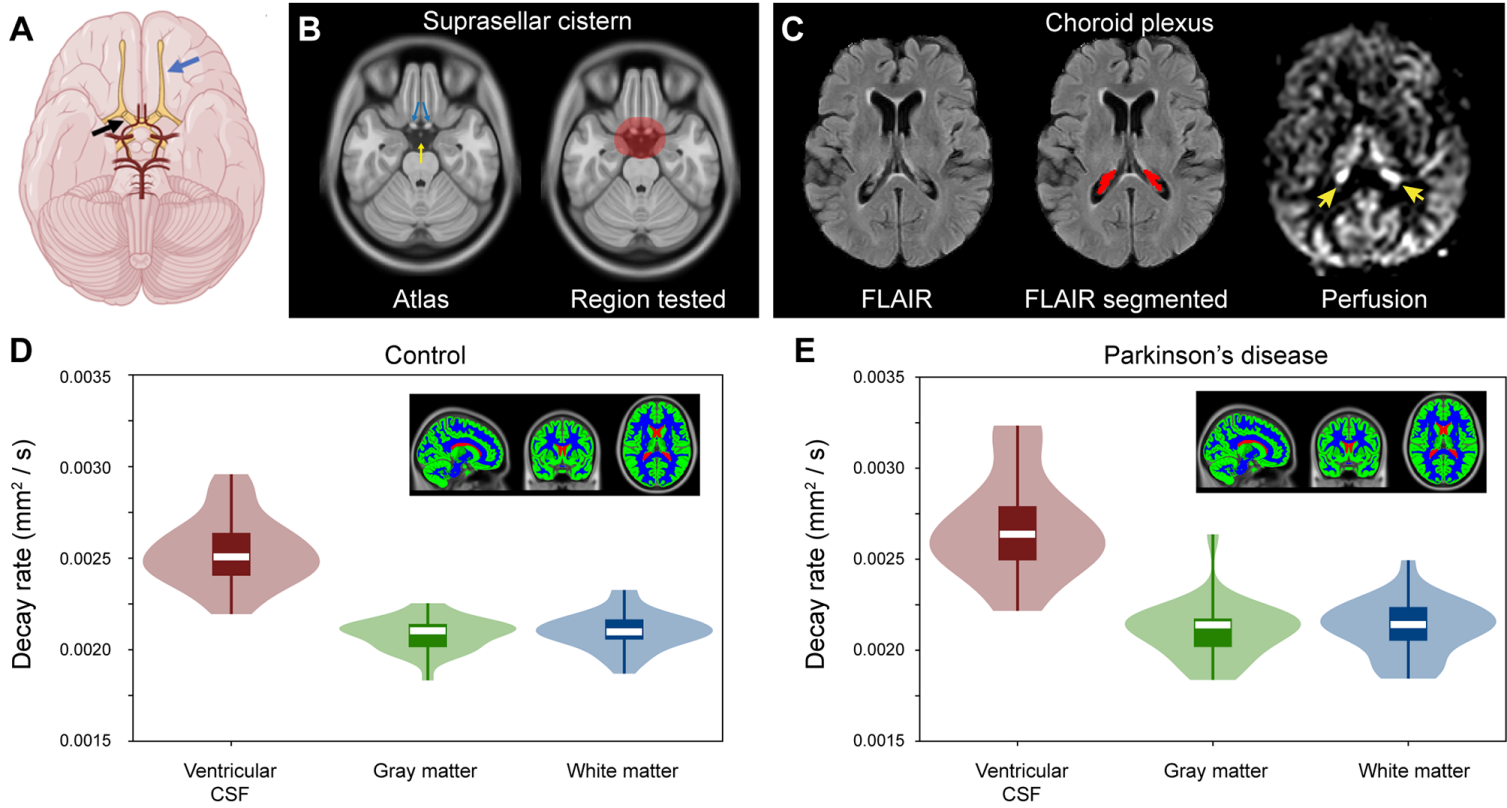
Localization and quantification procedures. (**A**) Suprasellar cistern location, which co-localizes with major cranial nerves including the olfactory (blue arrow) and optic (black arrow) nerves, along with circle of Willis (red vasculature). (**B**) The region of interest encompassing the suprasellar cistern from which spatial statistical testing was performed is overlaid on the standard 1 mm brain atlas; note that the region deliberately extended beyond the average suprasellar cistern boundaries to accommodate potential morphological variations. (**C**) Choroid plexus at the level of the lateral ventricles visible on FLuid Attenuated Inverstion Recovery (FLAIR) MRI, along with example of segmentation; the perfusion map is shown to right, which highlights (yellow arrows) the high choroid plexus perfusion signal which is comparable to gray matter perfusion signal. Given the high variability in choroid plexus anatomy, structures were segmented in native space for each participant using previously-reported machine learning routines (see *Methods*). (**D-E**) Across major tissue types (shown as inserts), decay rates are significantly elevated in CSF relative to other tissue types (*p* < 0.001), a well as in gray matter relative to white matter (*p* < 0.001). The plots show boxplots overlaid on violin plots. See Table 1 for quantitative values. No significant difference was observed between control and Parkinson’s disease participants for any tissue type

Figure 3A depicts the common suprasellar cistern region of interest considered for voxel-wise analysis, both in terms of the location relative to arteries of the circle of Willis and optic nerves. The common suprasellar cistern region-of-interest overlaid on the atlas and used for voxel-wise statistics in all participants is shown in Fig. 2B and an example of the deep learning algorithm applied to isolate the choroid plexus at the level of the lateral ventricles is shown in Fig. 2C. Note that given inter-subject variability in choroid plexus anatomy, this segmentation was performed for each participant. To confirm that the method could detect differences in regions with known fluid differences, signal decay in different tissue types was first considered. Figure 2D-E shows the gray matter, white matter, and CSF segmentation, along with violin and box plots of decay rates. Decay rates were significantly elevated (*p* < 0.001) in CSF relative to other tissues, as well as in gray matter (*p* < 0.001) compared to white matter. This finding was observed both in the PD and non-PD control participants and these global values did not differ between groups (Table 1).

**Fig. 4:**
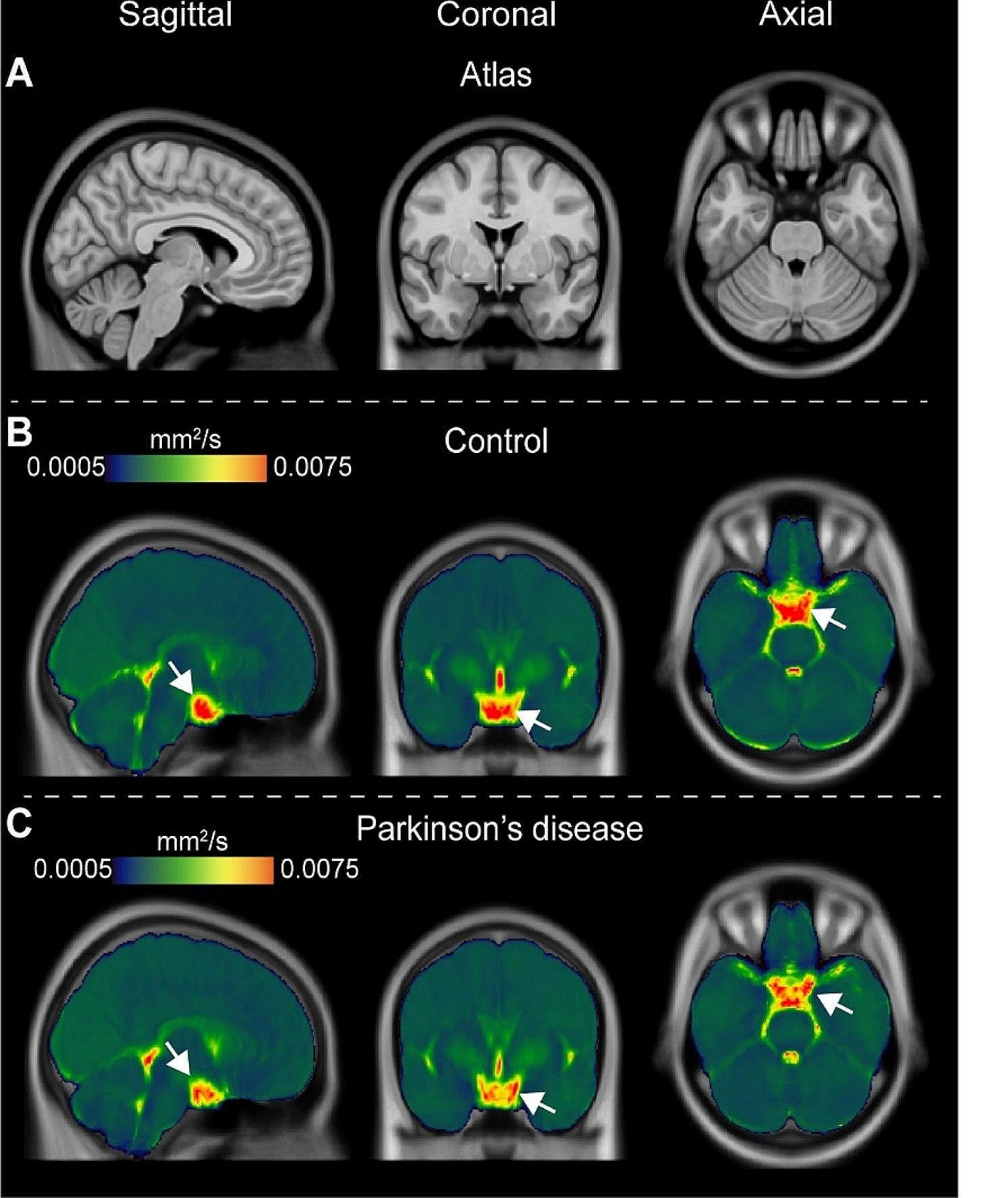
Group-averaged orthogonal maps of decay rates. Orthogonal representations are shown, for the level of the (**A**) *T*_1_-weighted atlas, (**B**) control participants, and (**C**) Parkinson’s disease (PD) participants. Reduced suprasellar flow is observed in PD relative to control participants. The changes are largely localized to the posterior region of the suprasellar cistern and pre-chiasmatic optic nerve. Sagittal representations across midline are shown separately in Fig. 5

**Fig. 5:**
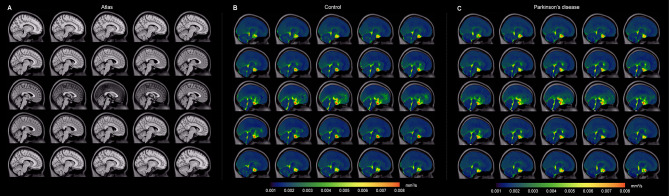
Group-averaged sagittal maps of decay rates. Group-averaged maps are shown sagittal across midline. Note that the scale has been changed slightly from that in Fig. 4 to show contrast of additional structures. The most prominent changes are appreciated in the posterior aspect of the suprasellar cistern and spaces along major neurofluid routes (e.g., fourth ventricle, cerebral aqueduct, and cisterns) with general similarity across cortex and other subarachnoid spaces

Figure 4 shows orthogonal representations of group level decay rate maps, separately for the non-PD control and PD participants. When group level values were considered in the suprasellar cistern, the decay rates were found to be significantly higher in control (*D* = 0.00673 ± 0.00213 mm^2^/s) relative to PD (*D* = 0.00517 ± 0.000110 mm^2^/s) participants (*p* < 0.001), consistent with the primary study hypothesis. We also observed that within the optic nerve the decay rates trended higher in control (*D* = 0.00453 ± 0.00249 mm^2^/s) relative to PD (*D* = 0.00363 ± 0.00108 mm^2^/s) participants (*p* = 0.004). There was no relationship observed for the decay rate and age (*p* = 0.342) or sex (*p* = 0.069) among PD and non-PD control participants on linear regression, however, this latter finding did not meet stated criteria for statistical significance. Figure 5 summarizes the group-averaged decay rates for both cohorts as sagittal representations around midline, which provides a more complete perspective on the spatial extent of decay rates and associated fluid motion between groups. Note that the scale has been adjusted slightly between Figs. 4 and 5 to better visualize contrast differences in different regions.

**Fig. 6:**
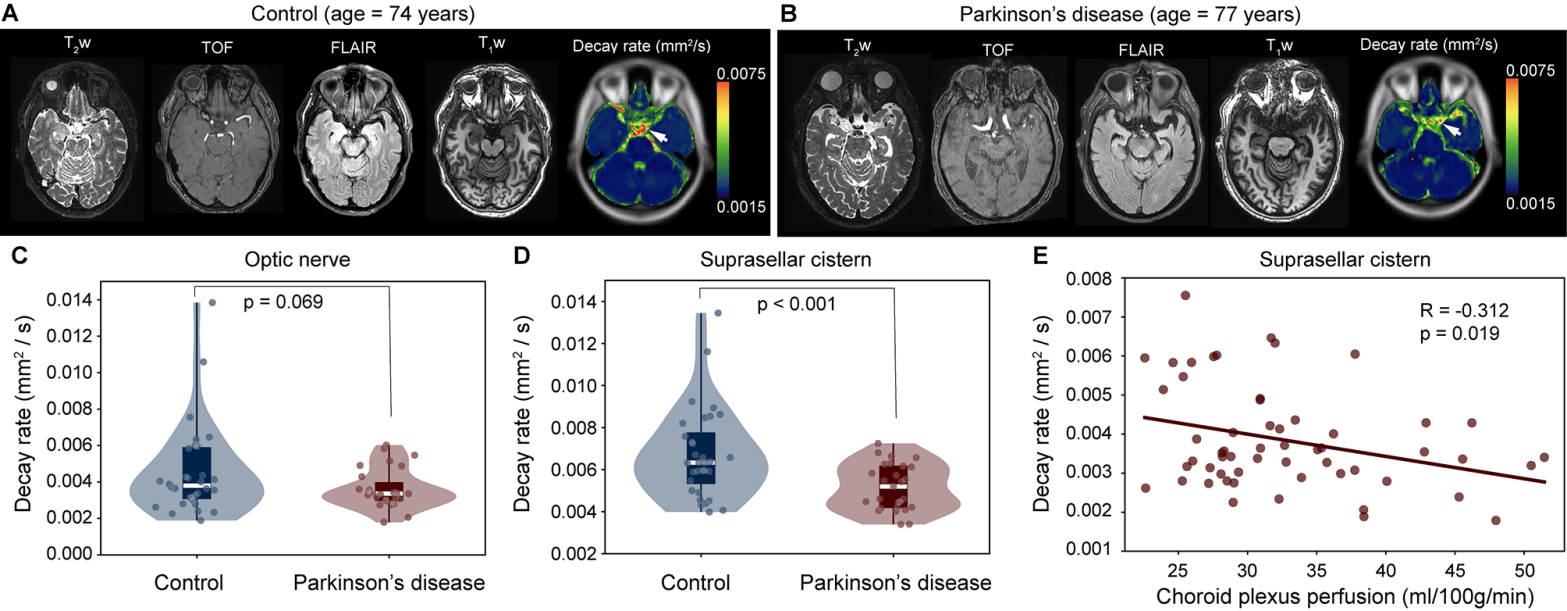
Case and group findings. Case example of an age- and sex-matched control (**A**) and Parkinson’s disease (PD) (**B**) participant showing anatomical imaging at the level of the suprasellar cistern. (**C-D**) Below, group level results are shown. Violin plots show the distribution of decay rates in control vs. PD participants, both in the suprasellar cistern and at the approximate location of the pre-chiasmatic optic nerve. The decay rates were significantly different in the suprasellar cistern (*p* < 0.001). (**E**) The decay rate was observed to inversely correlate with the choroid plexus perfusion in the suprasellar cistern (*p* = 0.019)

Figure 6 summarizes the distribution of decay rates across cohorts and against choroid plexus perfusion. Case examples which depict aspects of the group-level findings are shown in Fig. 6-B. We observed no significant difference in choroid plexus perfusion between groups (control: 32.7 ± 6.1 ml/100 g/min; PD: 32.8 ± 8.2 ml/100 g/min; *p* = 0.932); however, we observed an inverse correlation between choroid plexus perfusion and decay rate (Spearman’s *r* =-0.312; *p* = 0.019) across all participants in the suprasellar cistern region. As described above, all maps, tables, an data plots report decay rate values for the fitting that included a term to model the noise floor. Summary statistics from the fitting performance are shown in Table 2 across regions, which depict how the fitting performance changes with versus without this term included.

No significant relationship was observed between the decay ranges and either UPDRS score or MoCA score for any of the model fitting variants. Of note, none of the analyses above differed significantly with regard to whether the participant with MoCA < 24 was included and all results above report values from the full cohort.

## Discussion

Diffusion weighted imaging (DWI) MRI with diffusion weightings prescribed over the approximate kinetic regime of CSF motion within the suprasellar cistern was applied in adults with PD and age-matched non-PD controls. Significantly attenuated motion was observed in the suprasellar cistern of PD relative to control participants and the extent of motion attenuation was associated with increased choroid plexus perfusion. The methodology proposed, which is similar to an apparent diffusion constant calculation from low *b*-value DWI MRI, can be implemented on clinical scanners and could represent an additional non-invasive tool for assessment of fluid kinetics in the setting of neurodegeneration.

### Expansion of methodologies to assess fluid movement non-invasively in vivo

Modeling the diffusion coefficient in principle provides quantitative kinetic information and may present a candidate non-tracer technology for quantifying neurofluid movement non-invasively in vivo. Fitting results demonstrated that within fluid filled spaces (specifically the suprasellar cistern investigated here), *b*-values over an approximate range of 0–350 s/mm^2^ are adequate to assess this decay (e.g., Fig. 2) and the fidelity of fitting can be improved by modeling the noise floor (Table 2). This methodology should be considered in light of other MRI-based assessments of quantitative fluid movement. Most commonly, phase contrast magnetic resonance angiography, generally with velocity encoding gradients of 50–100 cm/s or higher can be used to quantify peak systolic arterial flow velocities in the major cervical vessels (velocity = 75–125 cm/s) [34] or intracranial vessels of the circle of Willis (70–160 cm/s) [35]. In major venous structures, peak systolic velocity is lower and within a healthy range of 10–35 cm/s for most major venous structures [36]. Similar phase contrast methods can be applied at the level of the cerebral aqueduct to assess CSF flow en route from the third to fourth ventricle, as peak CSF flow velocity is frequently reported as 10–20 cm/s [8–10]. However, these phase contrast approaches are generally not practical for assessing CSF movement in other structures given the much slower flow profiles in these regions. Under these physiological conditions, sufficiently low velocity encoding gradients are beyond the hardware limits of clinical scanners. Blood arrival to tissue, and even CSF flow [37] and peripheral lymphatic flow [38], have also been assessed using principles of spin labeling with multiple delay times. However, the duration of the generated magnetic label is governed by the *T*_1_ relaxation time of the water spins in the separate fluids, which, at 3 Tesla, is approximately 1.624s for blood water [25], 3.817s for CSF [39], and 3.100s for lymphatic fluid [38]. Therefore, principles of spin labeling can only be applied to assess fluid movement over short distances or when velocity is high.

This work utilizes principles of diffusion and the slower and more isotropic fluid motion in the suprasellar cistern to estimate CSF kinetics. The theory of estimating diffusion properties from different levels of diffusion weightings has been well characterized [40] and the approach proposed here is similar to a calculation of the apparent diffusion coefficient, albeit over a range of diffusion weightings relevant to CSF motion. Recent work has applied DWI at different *b*-values to qualitatively estimate fluid motion from categorical scoring of the rate of decay in ten healthy adults (aged = 25–58 years) in posterior fossa, suprasellar cistern, and Sylvian vallecula compared to the lateral ventricle and frontal and parietal CSF spaces [12]. This work also provided support for greater CSF fluid motion in the third ventricles in younger versus older participants, however, the method required radiological scoring and quantitative assessment was not proposed. Our work extends the approach to enable quantitative, continuous assessments of the decay rate.

### CSF motion within the suprasellar cistern

The suprasellar cistern is located at the level of the circle of Willis, superior to the sella turcica, anterior and inferior to the hypothalamus, and bordered by the uncus of the temporal lobes. Importantly, the suprasellar cistern is filled with freely circulating CSF and additionally contains the optic chiasm, the infundibular stalk, and the cerebrovascular circle of Willis.

Growing evidence suggests that pulsatility of the intracranial vasculature is fundamental to fluid motion within the cerebrum [41, 42]. Compliance of the cerebrovasculature and healthy pulsatility has been proposed to assist with movement of fluids within the cerebrum [6]. Patients with bilateral intracranial vasculopathy have been observed to have elevated perfusion of the choroid plexus, which may be required to upregulate CSF production activity in the presence of reduced pulsatility of arteries secondary to vasculopathy [43]. In support of this possibility, it has also been shown that choroid plexus perfusion reduces after successful angiogenesis-inducing indirect surgical revascularization in these patients [43], and also, that the choroid plexus activity may be reduced in the presence of high flow scenarios and sickle cell disease, where increases in cerebral blood volume are required to compensate for reduced blood oxygen content from anemia [18]. However, in this study we do not measure arterial pulsatility and as such additional work is required to investigate this possibility in PD.

The observation that CSF motion has high inter-subject variability in the cisterns has previously been shown in the intracranial vessel wall imaging literature [44], whereby delay-alternating-nutation-with-tailored-excitation fluid suppression modules [45] were utilized to null basal cistern CSF signal in a velocity-dependent manner, which when effective increase conspicuity of the intracranial vessel walls. However, the degree of fluid attenuation has been reported to be highly participant-dependent, a finding attributed to high variability of inter-subject neurofluid motion within the basal cisterns [44]. This work extends these findings to demonstrate that CSF motion within the suprasellar cistern varies between PD and age-matched non-PD adults, and interestingly, is inversely related to the perfusion of the choroid plexus. These findings taken together could be explained by the choroid plexus upregulating CSF production activity, or responding to circulating markers, in the presence of reductions in intracranial CSF motion; however, additional work is required to understand this relationship fully.

### CSF flow aberrations in Parkinson’s disease

There is a growing literature reporting aberrant CSF circulation in PD, as well as other neurodegenerative proteinopathies [46]. Specifically with regards to PD, dynamic contrast-enhanced magnetic resonance imaging has been applied to assess meningeal lymphatic flow in cognitively normal controls and patients with idiopathic PD or atypical Parkinsonian disorders, and it was observed that patients with idiopathic PD exhibited significantly reduced flow through the meningeal lymphatic vessels along the superior sagittal sinus and sigmoid sinus, as well as a notable delay in deep cervical lymph node perfusion, compared to patients with atypical Parkinsonian disorders [47]. These findings suggest that differences in fluid circulation may partly underlie differences between idiopathic and atypical Parkinsonian disorders. Furthermore, mice injected with α-synuclein preformed fibrils demonstrated delayed meningeal lymphatic drainage, loss of tight junctions among meningeal lymphatic endothelial cells and increased inflammation of the meninges [47].

Prior work using principles of diffusion to estimate water motion along perivascular spaces has provided evidence in support of reduced water motion along perivascular spaces in older adults with PD relative to essential tremor [2]. This finding was tentatively attributed to PD being a proteinopathy and that perivascular fluid motion aberrations may contribute to the retention of α-synuclein. This finding is partly consistent with other work: aquaporin-4 (AQP4) channels are likely essential for effective transport of fluid between perivascular and interstitial spaces, and separate work has shown that α-synuclein deposition negatively correlates with AQP4 expression in patients with PD [48]. Additionally, Fang et al. [49] evaluated AQP4 polymorphisms in PD together with Positron Emission Tomography measures of beta-amyloid burden, sleep behavior, and CSF biomarkers and observed that AQP4 rs162009 may be a biomarker of cognitive decline in PD, possibly attributable to its role in changing neurofluid circulation.

Finally, we observed no relationship between cognition, assessed with MoCA, and the imaging measures. However, this is not surprising given that most participants enrolled in this work had no or only very mild cognitive impairment. For motor impairment, assessed with UPDRS, we also observed no significant relationship between the metrics of CSF motion within the suprasellar cistern, however, the study was not powered to evaluate this relationship rigorously, which would require approximately 60 patients (given the effect sizes observed) and likely a multiple regression analysis with additional control for other relevant covariates such as age, sex, and disease duration. As the proposed method can be readily applied in 5–10 min, future work that assesses how CSF motion within the cisterns relates to symptomatology, together with other established markers of disease severity, may be useful.

### Limitations

First, the spatial resolution of typical DWI scans at the clinical field strengths of 3 Tesla is 8–27 mm^3^ (voxel volume), which is at or larger than the size of many structures relevant to perivascular flow. Therefore, we focused our study on the suprasellar cistern, which has a volume several orders of magnitude larger than the spatial resolution of the voxel. Since the suprasellar cistern contains the major arteries of the circle of Willis and components of cranial nerves implicated in fluid motion, and is comprised of freely circulating CSF, it represents a logical location for assessment of fluid motion. However, we did not attempt to interrogate smaller structures with emerging relevance to perivascular or potential interstitial flow (e.g., cribriform plate or parasagittal dural spaces). Nonetheless, partial volume effects, especially when contrasting CSF values between the suprasellar cistern and ventricles, which have different spatial extents, cannot be ruled out. Second, given limited information available on kinetic CSF motion within the suprasellar cistern, it remains unclear which velocity or diffusion regime is most relevant. Work by others in 50 healthy adults and 50 patients with ventricular dilation demonstrated that at the *b*-value of 500 s/mm^2^, signal voids in 3rd and 4th ventricles, the cerebral sulci and the Sylvian fissure were observed, thereby providing evidence for this range being reasonable for assessing CSF flow [50]. Therefore, we applied diffusion weightings 0–1000 s/mm^2^ and characterized the signal decay quantitatively with and without a term that incorporated a noise floor. We observed that the *b*-value range of approximately 0–350 s/mm^2^ is more appropriate for reliable fitting in the region of the suprasellar cistern (e.g., Fig. 2) and future work could benefit by oversampling this lower *b*-value regime while also modeling the noise floor, as was performed here. Finally, the absolute choroid plexus perfusion measures should be interpreted with caution. Recent studies have indicated that choroid plexus perfusion measured at short echo time comprises both perfusion and water exchange at the level of the blood-CSF barrier [51–53]. Therefore, the perfusion values reported in this study are reflective of apparent perfusion rather than absolute perfusion [9, 23].

In conclusion, diffusion weighted imaging with multiple low-to-intermediate *b*-values was applied to provide evidence supporting reduced CSF motion within the suprasellar cistern in participants with versus without Parkinson’s disease. The extent of attenuation of fluid movement corresponded with choroid plexus hyperemia at the level of the suprasellar cistern. These findings suggest that low *b*-value diffusion weighted imaging may provide a new imaging tool to interrogate CSF motion in the growing number of applications where CSF circulation dysfunction is implicated.

## Data Availability

Source imaging and demographic data will be made available to investigators with appropriate Collaborative of Institutional Training Initiative Responsible Conduct of Human Research and Good Clinical Practice training upon request.

## Abbreviations

ADC: Apparent diffusion coefficient
ANTs: Advanced Normalization Tools
AQP4: aaquaporin-4
CSF: Cerebrospinal fluid
DWI: Diffusion weighted imaging
FLAIR: FLuid Attenuated Inverstion Recovery
IRB: Institutional Review Board
MoCA: Montreal Cognitive Assessment
MRI: Magnetic resonance imaging
pCASL: Pseudocontinuous arterial spin labeling
PD: Parkinson’s disease
RBANS: Repeatable Battery for the Assessment of Neuropsychological Status
RMSE: Root mean squared error
TE: Echo time
TR: Repetition time
UPDRS: Unified Parkinson’s Disease Rating Scale

## References

[1] Bloem BR, Okun MS, Klein C (2021). Parkinson’s disease. Lancet.

[2] McKnight CD, Trujillo P, Lopez AM (2021). Diffusion along perivascular spaces reveals evidence supportive of glymphatic function impairment in Parkinson disease. Parkinsonism Relat Disord.

[3] Salman MM, Kitchen P, Iliff JJ, Bill RM (2021). Aquaporin 4 and glymphatic flow have central roles in brain fluid homeostasis. Nat Rev Neurosci.

[4] McKnight CD, Rouleau RM, Donahue MJ, Claassen DO (2020). The regulation of cerebral spinal fluid Flow and its relevance to the Glymphatic System. Curr Neurol Neurosci Rep.

[5] Ringstad G, Valnes LM, Dale AM et al. Brain-wide glymphatic enhancement and clearance in humans assessed with MRI. JCI Insight 2018;3(13). 10.1172/jci.insight.121537 PMC6124518 29997300

[6] Iliff JJ, Wang M, Zeppenfeld DM (2013). Cerebral arterial pulsation drives paravascular CSF-interstitial fluid exchange in the murine brain. J Neurosci.

[7] Shah T, Leurgans SE, Mehta RI et al. Arachnoid granulations are lymphatic conduits that communicate with bone marrow and dura-arachnoid stroma. J Exp Med 2023;220(2). 10.1084/jem.20220618 PMC9728136 36469302

[8] Hett K, McKnight CD, Eisma JJ (2022). Parasagittal dural space and cerebrospinal fluid (CSF) flow across the lifespan in healthy adults. Fluids Barriers CNS.

[9] Eisma JJ, McKnight CD, Hett K (2023). Choroid plexus perfusion and bulk cerebrospinal fluid flow across the adult lifespan. J Cereb Blood Flow Metab.

[10] Spijkerman JM, Geurts LJ, Siero JCW, Hendrikse J, Luijten PR, Zwanenburg JJM (2019). Phase contrast MRI measurements of net cerebrospinal fluid flow through the cerebral aqueduct are confounded by respiration. J Magn Reson Imaging.

[11] Iliff JJ, Lee H, Yu M (2013). Brain-wide pathway for waste clearance captured by contrast-enhanced MRI. J Clin Invest.

[12] Taoka T, Kawai H, Nakane T (2021). Diffusion analysis of fluid dynamics with incremental strength of motion proving gradient (DANDYISM) to evaluate cerebrospinal fluid dynamics. Jpn J Radiol.

[13] Postuma RB, Berg D, Stern M (2015). MDS clinical diagnostic criteria for Parkinson’s disease. Mov Disord.

[14] Goetz CG, Tilley BC, Shaftman SR (2008). Movement Disorder Society-sponsored revision of the Unified Parkinson’s Disease Rating Scale (MDS-UPDRS): scale presentation and clinimetric testing results. Mov Disord.

[15] Nasreddine ZS, Phillips NA, Bedirian V (2005). The Montreal Cognitive Assessment, MoCA: a brief screening tool for mild cognitive impairment. J Am Geriatr Soc.

[16] Randolph C, Tierney MC, Mohr E, Chase TN (1998). The repeatable battery for the Assessment of Neuropsychological Status (RBANS): preliminary clinical validity. J Clin Exp Neuropsychol.

[17] Nilsson C, Stahlberg F, Thomsen C, Henriksen O, Herning M, Owman C (1992). Circadian variation in human cerebrospinal fluid production measured by magnetic resonance imaging. Am J Physiol.

[18] Johnson SE, McKnight CD, Jordan LC (2021). Choroid plexus perfusion in sickle cell disease and moyamoya vasculopathy: implications for glymphatic flow. J Cereb Blood Flow Metab.

[19] Eisma JJ, McKnight CD, Hett K (2024). Deep learning segmentation of the choroid plexus from structural magnetic resonance imaging (MRI): validation and normative ranges across the adult lifespan. Fluids Barriers CNS.

[20] Avants BB, Tustison NJ, Song G, Cook PA, Klein A, Gee JC (2011). A reproducible evaluation of ANTs similarity metric performance in brain image registration. NeuroImage.

[21] Lu H, Donahue MJ, van Zijl PC (2006). Detrimental effects of BOLD signal in arterial spin labeling fMRI at high field strength. Magn Reson Med.

[22] Buxton RB, Frank LR, Wong EC, Siewert B, Warach S, Edelman RR (1998). A general kinetic model for quantitative perfusion imaging with arterial spin labeling. Magn Reson Med.

[23] Zhao L, Taso M, Dai W, Press DZ, Alsop DC (2020). Non-invasive measurement of choroid plexus apparent blood flow with arterial spin labeling. Fluids Barriers CNS.

[24] Alisch JSR, Kiely M, Triebswetter C (2021). Characterization of age-related differences in the human choroid plexus volume, Microstructural Integrity, and blood perfusion using multiparameter magnetic resonance imaging. Front Aging Neurosci.

[25] Lu H, Clingman C, Golay X, van Zijl PC (2004). Determining the longitudinal relaxation time (T1) of blood at 3.0 Tesla. Magn Reson Med.

[26] Alsop DC, Detre JA, Golay X (2015). Recommended implementation of arterial spin-labeled perfusion MRI for clinical applications: a consensus of the ISMRM perfusion study group and the European consortium for ASL in dementia. Magn Reson Med.

[27] Fonov V, Evans AC, Botteron K (2011). Unbiased average age-appropriate atlases for pediatric studies. NeuroImage.

[28] Avants BB, Epstein CL, Grossman M, Gee JC (2008). Symmetric diffeomorphic image registration with cross-correlation: evaluating automated labeling of elderly and neurodegenerative brain. Med Image Anal.

[29] Prah DE, Paulson ES, Nencka AS, Schmainda KM (2010). A simple method for rectified noise floor suppression: phase-corrected real data reconstruction with application to diffusion-weighted imaging. Magn Reson Med.

[30] Stejskal EO, Tanner JE (1965). Spin diffusion measurements: spin echoes in the Presence of a time-dependent field gradient. J Chem Phys.

[31] Coupe P, Mansencal B, Clement M (2020). AssemblyNet: a large ensemble of CNNs for 3D whole brain MRI segmentation. NeuroImage.

[32] Pugh EA, Kemp EC, van Dyck CH, Mecca AP, Sharp ES (2018). Alzheimer’s Disease Neuroimaging I. effects of normative adjustments to the Montreal Cognitive Assessment. Am J Geriatr Psychiatry.

[33] Brooks BL, Iverson GL, White T (2007). Substantial risk of Accidental MCI in healthy older adults: base rates of low memory scores in neuropsychological assessment. J Int Neuropsychol Soc.

[34] Lee W (2014). General principles of carotid doppler ultrasonography. Ultrasonography.

[35] Wang L, Xing Y, Li Y, Han K, Chen J (2014). Evaluation of flow velocity in unilateral middle cerebral artery stenosis by Transcranial Doppler. Cell Biochem Biophys.

[36] Canhao P, Batista P, Ferro JM (1998). Venous transcranial doppler in acute dural sinus thrombosis. J Neurol.

[37] Yamada S, Miyazaki M, Kanazawa H (2008). Visualization of cerebrospinal fluid movement with spin labeling at MR imaging: preliminary results in normal and pathophysiologic conditions. Radiology.

[38] Rane S, Donahue PM, Towse T (2013). Clinical feasibility of noninvasive visualization of lymphatic flow with principles of spin labeling MR imaging: implications for lymphedema assessment. Radiology.

[39] Lu H, Nagae-Poetscher LM, Golay X, Lin D, Pomper M, van Zijl PC (2005). Routine clinical brain MRI sequences for use at 3.0 Tesla. J Magn Reson Imaging.

[40] Yablonskiy DA, Sukstanskii AL (2010). Theoretical models of the diffusion weighted MR signal. NMR Biomed.

[41] Lindstrom EK, Ringstad G, Mardal KA, Eide PK (2018). Cerebrospinal fluid volumetric net flow rate and direction in idiopathic normal pressure hydrocephalus. Neuroimage Clin.

[42] Wen Q, Tong Y, Zhou X, Dzemidzic M, Ho CY, Wu YC (2022). Assessing pulsatile waveforms of paravascular cerebrospinal fluid dynamics within the glymphatic pathways using dynamic diffusion-weighted imaging (dDWI). NeuroImage.

[43] Johnson SE, McKnight CD, Lants SK (2020). Choroid plexus perfusion and intracranial cerebrospinal fluid changes after angiogenesis. J Cereb Blood Flow Metab.

[44] Cogswell PM, Siero JCW, Lants SK (2018). Variable impact of CSF flow suppression on quantitative 3.0T intracranial vessel wall measurements. J Magn Reson Imaging.

[45] Li L, Miller KL, Jezzard P (2012). DANTE-prepared pulse trains: a novel approach to motion-sensitized and motion-suppressed quantitative magnetic resonance imaging. Magn Reson Med.

[46] Rasmussen MK, Mestre H, Nedergaard M (2018). The glymphatic pathway in neurological disorders. Lancet Neurol.

[47] Ding XB, Wang XX, Xia DH (2021). Impaired meningeal lymphatic drainage in patients with idiopathic Parkinson’s disease. Nat Med.

[48] Hoshi A, Tsunoda A, Tada M, Nishizawa M, Ugawa Y, Kakita A (2017). Expression of Aquaporin 1 and Aquaporin 4 in the temporal neocortex of patients with Parkinson’s Disease. Brain Pathol.

[49] Fang Y, Dai S, Jin C (2021). Aquaporin-4 polymorphisms are Associated with Cognitive Performance in Parkinson’s Disease. Front Aging Neurosci.

[50] Taoka T, Naganawa S, Kawai H, Nakane T, Murata K (2019). Can low b value diffusion weighted imaging evaluate the character of cerebrospinal fluid dynamics?. Jpn J Radiol.

[51] Perera C, Harrison IF, Lythgoe MF, Thomas DL, Wells JA (2021). Pharmacological MRI with simultaneous measurement of cerebral perfusion and blood-cerebrospinal fluid barrier function using interleaved Echo-Time arterial spin labelling. NeuroImage.

[52] Lee H, Ozturk B, Stringer MS (2022). Choroid plexus tissue perfusion and blood to CSF barrier function in rats measured with continuous arterial spin labeling. NeuroImage.

[53] Petitclerc L, Hirschler L, Wells JA (2021). Ultra-long-TE arterial spin labeling reveals rapid and brain-wide blood-to-CSF water transport in humans. NeuroImage.

